# TLR4 Regulates the Proinflammatory Response and Myeloperoxidase Activity in Neutrophils During Interaction with *Sporothrix schenckii*

**DOI:** 10.3390/jof12100779

**Published:** 2026-10-17

**Authors:** Yolanda Romo-Lozano, Aylin Mayela Avila-Colis, Daniel Cervantes-García, Rodolfo González-Segovia, Estela Ruiz-Baca

**Affiliations:** 1 Centro de Ciencias Básicas, Universidad Autónoma de Aguascalientes, Av. Universidad No. 940, Aguascalientes 20100, Mexico; aylin.avila@outlook.es (A.M.A.-C.); dcervantesga@secihti.mx (D.C.-G.); rodolfo.gonzalezs@edu.uaa.mx (R.G.-S.); 2 Secretaría de Ciencia, Humanidades, Tecnología e Innovación, Ciudad de México 03940, Mexico; 3 Facultad de Ciencias Químicas, Universidad Juárez del Estado de Durango, Av. Veterinaria S/N, Durango 34120, Mexico; eruiz@ujed.mx

**Keywords:** myeloperoxidase, neutrophils, *Sporothrix schenckii*, TRL4

## Abstract

*Sporothrix schenckii* is a pathogenic fungus that triggers innate immune responses. Toll-like receptors (TLRs), such as TLR4, upon recognizing microbial components (including fungal ones), activate inflammatory and immune responses in cells. Meanwhile, in neutrophils, myeloperoxidase (MPO) is involved in oxidative mechanisms with antimicrobial activity. In this context, the aim of this study was to evaluate the role of TLR4 in the neutrophil response to *S*. *schenckii* and its potential relationship with MPO. To achieve this, human neutrophils, untreated or pre-treated with an anti-TLR4 antibody, were exposed for two hours to baseline conditions, lipopolysaccharide (LPS) stimulation, or *S*. *schenckii* conidia. Subsequently, viability, phagocytosis, MPO activity, and protein expression (TLR4, MPO) were assessed via Western blot. Additionally, gene expression (*TLR4*, *MPO*, *IL8*, *TNF*, *IL18*, *IL1B*) was evaluated using RT-qPCR, and IL-8 levels were quantified by ELISA. The results showed that *S*. *schenckii* conidia increased TLR4 and MPO expression in neutrophils. TLR4 blockade tended to reduce phagocytosis and fungal viability nonsignificantly while increasing *MPO* expression, altering cytokine production (increasing *IL8*, *IL18*, and *TNF*, and decreasing LPS-induced *IL1B*). These findings suggest that TLR4-mediated fungal recognition may modulate inflammation, MPO secretion, and cytokine release to control infection, highlighting its potential as an immunotherapeutic target.

## 1. Introduction

Neutrophils, also known as polymorphonuclear cells (PMNs), represent the most abundant cell type in human blood and constitute the first line of defense in the innate immune response against bacterial and fungal infections [1]. These cells undergo terminal differentiation in the bone marrow and enter the circulatory system with a short lifespan. Their default fate is spontaneous apoptosis unless they are rescued by microbial or inflammatory stimuli. Upon detecting chemotactic signals emitted at infection sites, neutrophils are among the first non-resident leukocytes to be recruited to affected tissues to contain the threat [2].

The main immunological mechanisms associated with neutrophils are phagocytosis, degranulation (release of the contents of primary, secondary, tertiary, and secretory vesicles, notably including myeloperoxidase (MPO) and neutrophil elastase (NE)). generation of reactive oxygen species (ROS; production of superoxide anion, hydrogen peroxide (H_2_O_2_), and hypochlorous acid (HOCl)), formation of neutrophil extracellular traps (NETs; networks composed of nuclear or mitochondrial DNA), and secretion of pro-inflammatory cytokines (interleukin (IL)-1β and tumor necrosis factor-alpha (TNF-α)) [3].

To be activated, as a first step, the cell must recognize the pathogen through a receptor. These are denominated pattern recognition receptors (PRRs) and, recognize highly conserved molecular structures in microorganisms, known as pathogen-associated molecular patterns (PAMPs), as well as damage associated molecular patterns (DAMPs) or chromatin-associated molecular patterns (CAMPs) [4]. PRRs include Toll-like receptors (TLRs), C-type lectin receptors (CLRs), RIG-I-like receptors (RLRs), and NOD-like receptors (NLRs), which detect conserved components of the fungal cell wall such as chitin, mannans, and glucans. Upon PRR–fungal PAMP binding, signaling cascades are activated that stimulate the production and secretion of inflammatory mediators, including cytokines and chemokines, thereby priming and recruiting additional immune cells to the infection site [5].

Once neutrophils phagocytose fungal cells, they deploy both oxidative and non-oxidative mechanisms to eliminate the pathogen [5]. The oxidative mechanisms of the respiratory burst in mature neutrophils are associated with the enzyme nicotinamide adenine dinucleotide phosphate oxidase (NADPH oxidase), which is bound to the phagosomal membrane bilayer; it is regulated through its assembly to produce reactive oxygen species (ROS) with antimicrobial activity, particularly the intraphagosomal superoxide anion and H_2_O_2_. The latter influences the activation of MPO to produce chloride with the consequent formation of HOCl [6].

Although MPO constitutes nearly 5% of total protein in human neutrophils, it is not strictly required to control infections that other antimicrobial mechanisms can eliminate [7]. However, its vital role in innate immunity becomes apparent when those alternative defense mechanisms prove to be insufficient or are evaded during pathogen challenge. Furthermore, extracellular MPO, which can be released following activation by inflammatory mediators (such TLR agonists), can modify inflammatory mediators, induce a proteolytic environment in the affected tissue, regulate apoptosis of other immune cells, and even be released into NETs [8].

Sporotrichosis is caused by dimorphic fungi species of the genus *Sporothrix,* among which *S*. *schenckii* stands out as one of the most widely studied. This mycosis affects both humans and animals, thus constituting a zoonosis of clinical relevance. It is mainly found in immunocompetent individuals following traumatic inoculation in skin by fungal propagules; those are present in plant material or soil and can lead to severe complications and disseminated infections in immunocompromised patients [9].

In sporothrichosis, the initial defense stage depends primarily on phagocytic cells, mainly neutrophils and mononuclear cells (monocytes and tissue-resident macrophages). In response to a *S*. *schenckii* infection, various PRRs participate in the initiation of the innate immune response associated with phagocytic cells. The cell wall of *S*. *schenckii* possesses a bilayered structure made of peptidorhamnomannan, chitin, β-1,3-glucan, β-1,4-glucan, and β-1,6-glucan; those structural components are recognized by different PRRs, including TLR2 and TLR4 [10]. TLR4 has been shown to recognize rhamnose-based oligosaccharides, which constitute key determinants for fungal virulence [11].

Numerous studies focus on the recognition of *Sporothrix* species via TLRs in multiple immune response cell lines; however, the specific role of the neutrophils is poorly known, even though these cells express a broad repertoire of PRRs [2]. Among these receptors, TLR2 and TLR4 have been actively implicated in the immunopathogenesis of sporotrichosis through phagocytosis and cytokine secretion in macrophages, although their precise role in neutrophils remains to be elucidated. In this regard, it has been demonstrated that the recognition of *S*. *schenckii* yeasts by TLR4 in macrophages induces the secretion of various pro-inflammatory cytokines such as the TNF-α [12], the production of nitric oxide (NO) [13]. Likewise, the cooperation of TLR2 and TLR4 for the production and secretion of effector cytokines such as IL-1β, has been evidenced when the peripheral blood mononuclear cells recognize the peptidorhamnomannan of *S*. *schenckii* and *S*. *brasiliensis* [14]. In other studies, only a slight overexpression of TLR4 was observed when keratinocytes interacted with *S*. *schenckii* [15]. In studies with *S*. *globosa*, it has been demonstrated that ROS production and NET formation occur with higher activity when the fungus interacts with high-density neutrophils (HDNs) than when it does with low-density neutrophils (LDNs); this is related to a hypo-inflammatory response and the low gene expression of PRR-related genes such as TLRs, NLRs, and RIG-I-like receptors [16].

Previous studies demonstrated that during an experimental sporotrichosis caused by *S*. *schenckii*, MPO activity is higher in infected animals and that it is primarily effective via neutrophils rather than macrophages [17]. Furthermore, the ROS production associated with MPO is, in part, responsible for the antifungal activity [18]. Because there is limited knowledge to understand the relationship between the recognition of *S*. *schenckii* by neutrophil TLR4 and MPO activity, this study aims to investigate the role of TLR4 in the regulation of the proinflammatory response and MPO activity in human neutrophils during their interaction with *S*. *schenckii*, thereby contributing to a deeper understanding of this innate immune mechanism.

## 2. Materials and Methods

### 2.1. Ethics Statement

The Institutional Bioethics Committee of the Universidad Autónoma de Aguascalientes (Mexico) approved this study (approval no. SG-1783-2023, project PIBB24-1, on 30 October 2023). All adult volunteers signed an informed consent form prior to blood donation for research purposes.

### 2.2. Fungal Strain and Culture Conditions

The *S*. *schenckii* sensu stricto wild-type strain UAA-307, isolated from a human lymphocutaneous sporotrichosis case, was used in all experiments. This strain was previously identified using morphology and the calmodulin gene (*CaM*) (GenBank accession number KJ921740) [19].

The fungus was cultured on Petri dishes with Sabouraud dextrose agar supplemented with antibiotics (cycloheximide and chloramphenicol) (SDA-A) (Becton, Dickinson and Company, BD, Sparks, MD, USA) for 3 days at 28 °C. Fungal biomass was harvested by gentle scraping, resuspended in sterile distilled water, filtered, and washed by centrifugation at 1096× *g* for 20 min at 4 °C. The resulting cell pellet was resuspended in phenol red-free Roswell Park Memorial Institute (RPMI)-1640 medium (Sigma-Aldrich, St. Louis, MO, USA; R8758) supplemented with 5% fetal bovine serum (FBS; Biowest/Microlab, Mexico City, Mexico; BAC62) and 1% penicillin/streptomycin (Sigma-Aldrich, St. Louis, MO, USA). Conidia quantification and cell viability were assessed in a Neubauer chamber using trypan blue exclusion assay, selecting suspensions with >98% viability. To obtain heat-killed conidia, aliquots of the suspensions were incubated in a water bath at 60 °C for 2 h; cell death was confirmed by the complete absence of colony-forming units (CFUs) on Sabouraud dextrose agar (SDA) (Becton, Dickinson and Company, BD, Sparks, MD, USA) across repeated assays.

### 2.3. Neutrophils Isolation

Heparinized venous blood was obtained from healthy donors. For each independent experiment, blood from two different donors was used, which generally varied across experimental replicates. Cells were purified using a discontinuous density gradient consisting of Lymphoprep™ (Serumwerk Bernburg, AG, Bernburg, Germany; 1114544) and Histopaque^®^-1119 (Sigma-Aldrich, St. Louis, MO, USA; 11191) centrifuged at 1397× *g* (Centrifuge Sigma 2-5, Sigma Laborzentrifugen GmbH, Osterode am Harz, Germany), as previously described [20]. Residual erythrocytes were lysed by osmotic shock using a 0.15 M NH_4_Cl solution. Following isolation, purified neutrophils from both donors were pooled for each independent assay, were washed twice with phosphate-buffered saline (PBS, pH 7.2) and resuspended in RPMI-1640 medium for cell counting, viability, and purity assessments. Cell concentration was determined using a Neubauer chamber, and neutrophil viability was evaluated using the Trypan blue exclusion assay. Only preparations with neutrophils purity and viability exceeding 90% were used in subsequent experiments.

### 2.4. Human Neutrophil–S. schenckii Interaction Assays

Neutrophils were permitted to rest at 37 °C for 30 min in an initial volume of 100 µL of RPMI-1640 to allow for cellular stabilization. Where indicated, cells were preincubated with a neutralizing anti-TLR4 antibody added in a volume of 100 µL (1:100; Mouse Monoclonal Antibody [HTA125], Invitrogen, Thermo Fisher Scientific, Waltham, MA, USA; MA5-16719) for 30 min at 37 °C, while cells without blocking received 100 µL of RPMI-1640. Cells were then exposed to the corresponding treatments added in a volume of 50 µL: stimulation with lipopolysaccharide (LPS; evaluated at 0, 200, 300, 400, and 500 ng) from *Escherichia coli* O111:B4 (Sigma-Aldrich, St. Louis, MO, USA; L2630) as a positive control, challenge with *S*. *schenckii* conidia, or medium to complete the volume in the control groups. This general protocol was followed across all experiments; however, the absolute number of neutrophils and conidia varied depending on the specific assay, maintaining a constant multiplicity of infection (MOI) of 1:5 (neutrophil-to-fungus ratio) where applicable. Interactions were carried out for 2 h at 37 °C in 1.5 mL polypropylene microcentrifuge tubes, reaching a final reaction volume of 250 µL of RPMI-1640, and reactions were terminated by chilling at 4 °C.

### 2.5. Neutrophil Viability Assay by MTT

Neutrophil viability (2 × 10^6^ cells) was determined by the conversion of the water-soluble MTT compound (Thiazolyl Blue Tetrazolium Bromide; Sigma-Aldrich, St. Louis, MO, USA; M2128) into an insoluble formazan product. Neutrophils stimulated with LPS (200 ng) or with 10 × 10^6^ viable or heat-killed conidia, as well as non-stimulated neutrophils (2 × 10^6^ cells), with or without TLR4 blockade, were placed in 1.5 mL polypropylene microcentrifuge tubes in a final volume of 250 µL and incubated at 37 °C. After 2 h, supernatants were removed by centrifugation at 202× *g* for 10 min at 4 °C. Cells were then incubated with 100 µL of MTT solution (0.5 mg/mL) for 4 h at 37 °C. The formazan crystals produced by viable cells were dissolved in 200 µL of 0.1 N acid-isopropanol (2-propanol with HCl; Sigma-Aldrich, St. Louis, MO, USA; I9516). Samples were centrifuged at 17,531× *g* for 20 min at 4 °C to remove cell debris and fungal cells, and 100 µL of each supernatant was transferred to a 96-well plate. The optical density (OD) was measured at 595 nm using a microplate reader (iMark™ Microplate Absorbance Reader, Model 550, Bio-Rad Laboratories, Tokyo, Japan). Cell viability was expressed as a percentage and calculated using the following formula: Cell viability (%) = (OD of the test sample/OD of the control sample) × 100.

### 2.6. Determination of Phagocytic Activity by Optical Microscopy

To evaluate the effect of TLR4 blockade on phagocytosis the percentage of phagocytosing neutrophils was quantified by optical microscopy. Neutrophils (1 × 10^6^ cells) and conidia (5 × 10^6^) were co-cultured for 2 h in a final volume of 250 μL of RPMI-1640 in 1.5 mL microcentrifuge tubes at 37 °C. Following the incubation period, the cell pellet was harvested by centrifugation at 202× *g* for 10 min at 4 °C and gently resuspended in 30 μL of cold RPMI-1640. Smears prepared on sterile, degreased slides were fixed with absolute methanol, stained with Wright–Giemsa (Golden Bell; Zapopan, Jal., Mexico; 64050), and examined under an optical microscope (Motic BA310, Motic Instruments Inc., Hong Kong, China). Images were captured using an adapted digital camera and analyzed using the Motic Images Plus 2.0 software (v2.0; Motic Instruments Inc. Hong Kong, China). For each experimental condition, 200 cells were counted per slide to determine the percentage of neutrophils that had engulfed fungal cells [20]. Control groups consisted of neutrophils with or without TLR4 blockade in the absence of conidia.

### 2.7. Fungal Viability Assay by Colony-Forming Units (CFUs)

To evaluate the effect of TLR4 blockade in neutrophils on *S*. *schenckii* conidia viability, neutrophil-fungus co-cultures were prepared at a 1:5 ratio (1 × 10^6^ neutrophils to 5 × 10^6^ conidia), both with and without TLR4 blockade. Following a 2 h interaction period, the samples were centrifuged at 1096× *g* for 20 min at 4 °C (Hermle Z216MK centrifuge, HERMLE Labortechnik GmbH, Wehingen, Germany; 61125015) to harvest the fungal cells. The resulting pellet was resuspended by inversion in 1 mL of sterile distilled water adjusted to pH 11 with NaOH [21] and incubated for 5 min at 37 °C to induce neutrophil lysis. To ensure the complete release of phagocytosed conidia, the samples were vigorously vortexed for 10 s (Vortex-Genie 2, Daigger, Modelo G560, Bohemia, NY, USA), followed by the preparation of ten-fold serial dilutions.

For the 1:10^4^ dilution, a 100 μL aliquot was plated onto SDA dishes using the spread-plate method. After an incubation period of up to 4 days at 28 °C, colony-forming units (CFUs) were quantified, and results were expressed as a percentage of the CFUs obtained from control samples (*S*. *schenckii* conidia not challenged with neutrophils) incubated under identical conditions. Additionally, 10 μL aliquots from each serial dilution and the initial stock suspension were inoculated onto SDA dishes using the drop-plate method.

### 2.8. Colorimetric Method for the Determination of Myeloperoxidase Activity

To evaluate the effect of TLR4 blockade on the myeloperoxidase (MPO) activity, neutrophils (1 × 10^6^ cells) challenged with *S*. *schenckii* (5 × 10^6^ conidia) were processed using 3,3′,5,5′-tetramethylbenzidine (TMB; Sigma-Aldrich, St. Louis, MO, USA; 860336) as a substrate, according to the modified method by Pulli et al. [22].

Following a 2 h interaction period, samples were centrifuged at 202× *g* for 10 min at 4 °C to separate the supernatant from the cell pellet. This first supernatant was subsequently centrifuged at 17,531× *g* for 20 min at 4 °C to obtain a cell-free fraction for the determination of secreted MPO activity.

Meanwhile, the cell pellet was lysed by vortexing (Vortex-Genie 2, Daigger, Model G560, Bohemia, NY, USA) in 300 μL of a 0.5% Triton X-100 solution in PBS (pH 7.2). After incubating on ice for 15 min, the samples were centrifuged at 17,531× *g* for 20 min at 4 °C to yield a second cell- and debris-free supernatant for quantifying residual (intracellular) MPO activity. The lysis treatment does not affect viability nor lyses in *S*. *schenckii* conidia.

For enzymatic activity quantification, 30 μL of each supernatant was loaded into a 96-well plate and mixed with 110 μL of a freshly prepared TMB working solution (2.9 mM TMB in 14.5% dimethyl sulfoxide (DMSO; Sigma, St. Louis, MO, USA; D2650) and 150 mM sodium phosphate buffer, pH 5.4). The reaction was immediately initiated by adding 80 μL of a 0.75 mM H_2_O_2_ solution (Hydrogen peroxide 30% RA ACS, J.T.Baker, Avantor, Radnor, PA, USA; 2186-01). Plates were incubated at 37 °C for 5 min, and the reaction was stopped with 50 μL of a 2 M H_2_SO_4_ solution. Finally, OD was measured at 450 nm using a microplate reader (iMark™ Microplate Absorbance Reader, Model 550, Bio-Rad Laboratories, Tokyo, Japan). RPMI-1640 medium and the lysis buffer used were included as blanks.

### 2.9. Immunofluorescence Assay

Human neutrophils (5 × 10^5^ cells) were seeded onto sterile round glass coverslips, placed in a 24-well plate, and incubated for 30 min at 37 °C in 100 μL of RPMI-1640 to allow cell adhesion. Where indicated, cells were subjected to TLR4 blocking for 30 min and then stimulated with either LPS (200 ng/250 μL RPMI-1640) or *S*. *schenckii* conidia (2.5 × 10^6^) in a final volume of 250 μL of RPMI-1640. After a 2 h incubation at 37 °C in a humidified atmosphere containing 5% CO_2_, the medium was carefully removed, and the cells were fixed with 4% paraformaldehyde (PFA) (Sigma-Aldrich, Milwaukee, WI, USA; P6148) in PBS (pH 7.2) for 30 min. Fixed cells were washed twice with PBS and permeabilized with 0.02% Triton X-100 in PBS. Nonspecific binding sites were blocked with 2% FBS and 0.02% Triton X-100 in PBS. For double immunodetection, cells were incubated overnight at 4 °C with polyclonal rabbit anti-MPO (1:200; DAKO, Agilent Technologies, Santa Clara, CA, USA; A0398) and mouse anti-TLR4 (1:100; Santa Cruz Biotechnology, Dallas, TX, USA; sc-293072) primary antibodies, diluted in PBS containing 0.02% (*v*/*v*) Triton X-100 and 3% bovine serum albumin (BSA; Equitech-Bio, Inc., Kerrville, TX, USA; BAC62). Samples were then washed and incubated for 2 h at room temperature with the secondary antibodies: Alexa Fluor™ 488 goat anti-rabbit IgG (H + L) (1:1000; Cross-Adsorbed Secondary Antibody, Invitrogen, Carlsbad, CA., USA; A-11008) and Alexa Fluor^®^ 594 goat anti-mouse IgG (H + L) (1:1000; Abcam, Waltham, MA, USA; ab150116), both diluted in PBS containing 3% FBS and 0.02% Triton X-100. DNA staining was performed with Hoechst 33258 (1:1000; Invitrogen, Carlsbad, CA., USA; H1398). Finally, coverslips were mounted using Mowiol mounting medium (Mowiol 4–88, Sigma-Aldrich, St. Louis, MO, USA; 81381) and examined by epifluorescence microscopy (Axioskop 40, CarlZeiss Microscopy, LLC, Thornwood, NY, USA), equipped with a CoolSNAP-Pro digital camera (Media Cybernetics, Inc., Rockville, MD, USA). Images were processed using Image-Pro Plus version 4.5.0.19 (Media Cybernetics, Rockville, MD, USA). Samples incubated without primary antibodies served as negative controls.

### 2.10. Western Blot Analysis

Human neutrophils (3 × 10^6^ cells) were incubated with or without TLR4-blocking antibodies and then stimulated with LPS or *S*. *schenckii* conidia (15 × 10^6^) for 2 h at 37 °C. Afterward, cells were centrifuged at 202× *g* for 10 min at 4 °C (Hermle Z216MK centrifuge, HERMLE Labortechnik GmbH, Wehingen, Germany, 61125015). Cell pellets were lysed with 30 μL of Radioimmunoprecipitation assay (RIPA) Lysis Buffer System (Santa Cruz Biotechnology, Dallas, TX, USA, c-24948) supplemented with protease inhibitors (200 nM phenylmethylsulfonyl fluoride [PMSF], 100 mM sodium orthovanadate, and a protease inhibitor cocktail). The mixture was incubated at 4 °C for 40 min under orbital agitation (Sea Star™ Digital Orbital Shaker; Heathrow Scientific, LLC, Vernon Hills, IL, USA) and then centrifuged at 17,531× *g* for 20 min at 4 °C. Protein concentration in the supernatant was determined using the Bradford assay against a BSA standard curve at 595 nm using a spectrophotometer (JENWAY 7305 UV/Visible Spectrophotometer; Cole-Parmer, Staffordshire, UK).

Protein samples (30 μg) were resolved by 10% SDS-PAGE and transferred onto membranes. Membranes were blocked with 5% skimmed milk (Svelty 0%, Nestlé, Mexico City, Mexico) and incubated overnight at 4 °C with the following primary anti-human antibodies: rabbit polyclonal anti-MPO (1:250; DAKO, Agilent Technologies, Santa Clara, CA, USA, A0398), rabbit polyclonal anti-TLR4 IgG (1:100; Invitrogen, Thermo Fisher Scientific, Waltham, MA, USA; 48-2300), rabbit polyclonal anti-Caspase-1 (ab1) IgG (1:500; Sigma-Aldrich, St. Louis, MO, USA; PRS3459), and rabbit polyclonal anti-*β*-actin IgG (1:500; Abcam, Waltham, MA, USA; ab8227) as a loading control. Membranes were then incubated with a horseradish peroxidase (HRP)-conjugated secondary antibody (1:500; Goat Anti-Rabbit IgG (H + L)-HRP Conjugate, Bio-Rad Laboratories, Hercules, CA, USA; 1706515). Protein bands were detected by chemiluminescence using Clarity Western ECL Substrate (Bio-Rad Laboratories, Hercules, CA, USA; 170-5060) and visualized using a C-DiGit Blot Scanner (LI-COR Biosciences, Lincoln, NE, USA, CDG-003787) with Image Studio™ Software (version 1.1.4; LI-COR Biosciences, Lincoln, NE, USA). Densitometric analysis was performed using ImageJ software (version 1.52h, National Institutes of Health, Bethesda, MD, USA). Protein expression levels were normalized to the corresponding β-actin loading control and expressed as relative mean band intensity.

### 2.11. Quantitative Real-Time PCR

Following the interaction of neutrophils (2 × 10^6^) with *S*. *schenckii* conidia (10 × 10^6^), cells were pelleted by centrifugation (202× *g*, 10 min). Total RNA was extracted using TRIzol reagent (Sigma-Aldrich, St. Louis, MO, USA; T9424). RNA integrity was verified by 2% agarose gel electrophoresis, Bio-Rad Laboratories, Inc., Hercules, CA, United States; 161-3101) in 1× TBE buffer (pH 8.3) with 10% SDS (Bio-Rad Laboratories, Inc., Hercules, CA, USA, 161-0301) and Blue/Orange 6X Loading Dye (sigma, Madison, WI, USA; G190A), visualized with 0.05% ethidium bromide on a Bio-Rad Universal Hood II Gel Doc System (Quantity One Software v4.6.8, Bio-Rad Laboratories, Inc., Hercules, CA, USA) against a 100 bp DNA ladder (Promega, Madison, WI, USA; G2101). RNA concentration and purity were measured using a Colibri Microvolume Spectrophotometer (Titertek Berthold, Pforzheim, Germany); samples with A_260_/A_280_ ≥ 1.6 were retained. Total RNA (1 μg) was reverse-transcribed according to the manufacturer’s instructions (RevertAid First Strand cDNA Synthesis Kit, Thermo Scientific, Waltham, MA, USA; K1622). Quantitative real-time PCR (qPCR) was performed on a StepOne™ Real-Time PCR System (Thermo Scientific, Waltham, MA, USA) using GoTaq qPCR Master Mix (Promega, Madison, WI, USA; A6001). Each 10 μL reaction contained 5 μL Master Mix, 0.5 μM forward/reverse primers (1 μL each), 50 ng cDNA, 0.2 μL CXR Reference Dye (300 nM), and 0.9 μL nuclease-free water. Thermal cycling parameters were as follows: 95 °C for 3 min, followed by 40 cycles of 95 °C for 30 s and 60 °C for 30 s, with a final melting curve step (60 °C to 95 °C with a 0.5 °C ramp). Target genes included *TLR4*, *MPO*, *IL8*, *IL18*, *TNF*, and *IL1B* (Table 1). Relative fold changes were calculated using the 2^−ΔΔCt^ method. Briefly, the ΔCt value was first calculated by subtracting the Ct of the reference gene (*GAPDH*) from the Ct of the target gene. Next, the ΔΔCt value was determined by subtracting the ΔCt of the control sample from the ΔCt of the treated sample. Finally, the fold change in gene expression was calculated using the 2^−ΔΔCt^ formula to evaluate the effects of each treatment on the expression of *IL8*, *IL1B*, *IL18*, *TNF*, *TLR4*, and *MPO*, with *GAPDH* serving as the internal reference gene. The primer sequences are listed in Table 1.

### 2.12. IL-8 Quantification by ELISA

Cell-free supernatants obtained from samples processed for RNA extraction and stored at −80 °C were used for IL-8 quantification using an enzyme-linked immunosorbent assay (ELISA) kit (Invitrogen IL-8 Human ELISA Kit, Thermo Fisher Scientific, Carlsbad, CA, USA; KHC0081), according to the manufacturer’s instructions. Optical density (OD) was measured at 450 nm using a microplate reader (iMark™ Microplate Absorbance Reader, Model 550, Bio-Rad Laboratories, Tokyo, Japan). Results were expressed in pg/mL and calculated against a standard concentration curve.

### 2.13. Statistical Analysis

Data were analyzed using a one-way ANOVA followed by Tukey’s post-hoc test for multiple comparisons or by an unpaired two-tailed Student’s *t*-test for two-group comparisons. Results are expressed as the mean ± standard error of the mean (SEM). Statistical significance was set at *p* < 0.05. All analyses were performed using GraphPad Prism software (version 8.01; GraphPad Software, Boston, MA, USA). All experiments were performed in at least two independent essays with technical duplicates, unless otherwise stated. When data correspond to a single experiment, this is indicated where necessary. For qualitative analyses or representative images, data from a single representative experiment are shown.

## 3. Results

### 3.1. Effect of LPS on Human Neutrophil Activation: Viability and MPO Activity

To select an optimal positive control for *S*. *schenckii* challenges, human neutrophils were stimulated with increasing LPS concentrations (0–500 ng/250 µL), a major component of the cell wall of Gram-negative bacteria and a PAMP that induces cellular responses via TLR4. Cell viability remained non-significantly altered across all concentrations (with an overall mean of 81.36 ± 17.53%, *p* > 0.05) (Figure 1A). Pro-caspase-1 expression was detected under all conditions; however, when challenged with both 200 and 400 ng/250 µL LPS as well as with conidia, its expression decreased, likely because it was converted into its active form—a hypothesis that remains to be demonstrated (Figure 1B). Residual intracellular (Figure 1C) and secreted (Figure 1D) MPO activity showed a non-significant dose-dependent decrease. Consequently, a 200 ng/250 µL LPS was selected for subsequent experiments as it significantly triggered MPO secretion without compromising viability.

### 3.2. Effect of TLR4 Blockade on TLR4 Protein Expression and Human Neutrophil Viability

Once the LPS concentration was established for use as a positive control for stimulation, TLR4 protein expression and viability were evaluated in neutrophils with and without TLR4 blockade. The receptor remained detectable across all treatments (Figure 2A), and blockade had no significant impact on cell viability (Figure 2B). Comparing unblocked neutrophils (viability set at 100%) to blocked neutrophils (98.73 ± 5.619%, *p* > 0.05), the decrease was minimal. Furthermore, viability remained above 90% across all other treatments, except for unblocked neutrophils interacting with heat-killed conidia (80.77 ± 24.56%), whose viability increased upon TLR4 blockade (109.8 ± 9.234%, *p* > 0.05). Notably, heat-killed conidia were used in the viability assay to rule out the possibility that any observed increase during interaction with viable conidia was due to fungal metabolic activity rather than neutrophil activity alone. Finally, the densitometric analysis showed non-significant increases in TLR4 expression across all challenged conditions compared to unblocked controls (Figure 2C).

To complement the evaluation of TLR4 expression in neutrophils following receptor blockade, dual immunofluorescence was performed (Figure 3). This technique confirmed the presence and localization of TLR4 (red), which remained evident regardless of whether the receptor was blocked or unblocked. Additionally, MPO expression (green) was observed in the cytoplasm of neutrophils. It should be noted that this qualitative assay primarily confirms the presence of these two cellular components rather than directly measuring the inhibitory effect of the blockade. Although thorough quantitative analyses are still required to definitively assess NET formation under both conditions, it was preliminarily observed that NET formation during the interaction with *S*. *schenckii* conidia appeared to be reduced in TLR4-blocked neutrophils compared to unblocked cells.

### 3.3. TLR4 Blockade on Neutrophils Decreases Phagocytosis and Viability of S. schenckii

Neutrophil TLR4 blockade significantly decreased the percentage of phagocytic cells compared to untreated neutrophils (44.00 ± 2.796% vs. 67.63 ± 4.706%, respectively; *p* < 0.05) (Figure 4A). Concurrently, a non-statistically significant reduction in fungal viability was observed in *S*. *schenckii* exposed to TLR4-blocked neutrophils versus control neutrophils (72.83 ± 5.610% vs. 87.49 ± 4.325%, respectively; *p* > 0.05) (Figure 4B).

### 3.4. Effect of TLR4 Blockade on Myeloperoxidase in Human Neutrophils Challenged with S. schenckii

To evaluate whether the TLR4 receptor is involved in the MPO response during the interaction between human neutrophils and *S*. *schenckii*, both MPO protein expression and enzymatic activity were measured across the different treatments.

Protein expression of the MPO (Figure 5A) showed only a slight decrease under TLR4 blockade conditions compared to non-blocked neutrophils, although this difference was not statistically significant (1.140 ± 0.2485 vs. 1.416 ± 0.3203, respectively; *p* > 0.05) (Figure 5B).

However, TLR4 blockade significantly increased intracellular (residual) MPO activity compared to non-blocked conditions. This increase was observed both upon stimulation with LPS (0.5784 ± 0.1414 vs. 0.1413 ± 0.02485, respectively; *p* < 0.001) and during challenge with *S*. *schenckii* conidia (0.5078 ± 0.08795 vs. 0.1904 ± 0.02107, respectively; *p* < 0.01). Likewise, in unstimulated neutrophils, TLR4 blockade doubled intracellular activity (0.4240 ± 0.1087) relative to the non-blocked control (0.2158 ± 0.03347), though this change did not reach statistical significance (*p* > 0.05) (Figure 5C).

These findings align with the lower secreted MPO activity observed in the extracellular medium following receptor blockade (Figure 5D). This reduction in secreted MPO was more pronounced in neutrophils challenged with LPS (0.3095 ± 0.04678 vs. 0.06160 ± 0.01349, respectively; *p* < 0.01) than with conidia (0.2736 ± 0.04848 vs. 0.1206 ± 0.04857, respectively; *p* > 0.05).

Finally, when comparing intracellular versus secreted MPO activity, statistically significant differences were observed across all assays where TLR4 was blocked (The greatest differences occurred under LPS stimulation (0.5784 ± 0.1414 vs. 0.06160 ± 0.01349; *p* < 0.001), followed by conidia challenge (0.5078 ± 0.08795 vs. 0.1206 ± 0.04857; *p* < 0.01), and unstimulated basal neutrophils (0.4240 ± 0.1087 vs. 0.04400 ± 0.01232; *p* < 0.05).

As a whole, these results suggest that blocking the TLR4 receptor inhibits MPO secretion, retaining enzymatic activity within the intracellular compartment and potentially affecting downstream neutrophil effector function; but specific tests are necessary to demonstrate it.

### 3.5. TLR4-Dependent Cytokine Gene Expression in Neutrophils Stimulated by Sporothrix schenckii

Relative *TLR4* gene expression (Figure 6A) increased in neutrophils stimulated with *S*. *schenckii* conidia; however, this increase was significantly higher in the presence of the receptor-blocking antibody (10.75 ± 2.13 vs. 4.32 ± 1.32, *p* < 0.05). In contrast, *TLR4* expression following blockade decreased in LPS-stimulated cells compared to those challenged with *S*. *schenckii* conidia (1.40 ± 0.25 vs. 10.75 ± 2.13, *p* < 0.001), as well as relative to the LPS-stimulated group without blockade (3.30 ± 0.92, *p* > 0.05).

Unlike the overexpression observed for *TLR4* and *MPO* gene expression (Figure 6B) decreased by approximately 49.2% (two-fold) upon receptor blockade in conidia-challenged neutrophils when compared to the unblocked stimulated group (1.593 ± 0.043 vs. 3.236 ± 0.395, respectively; *p* < 0.05). Likewise, stimulation with conidia induced a significant increase in *MPO* expression compared to the unstimulated control cells (3.236 ± 0.395 vs. 1.000 ± 0.016, *p* < 0.01).

Regarding cytokine production, *IL8* expression (Figure 6C) tended to increase following TLR4 blockade in conidia-stimulated neutrophils, although this difference was not statistically significant compared to the unblocked group (2.916 ± 0.332 vs. 2.139 ± 0.548, *p* > 0.05). Conversely, *TNFα* transcript levels (Figure 6D) increased significantly upon receptor blockade, both in LPS-stimulated cells (5.792 ± 0.889, *p* < 0.001) and in those challenged with conidia (6.795 ± 0.365, *p* < 0.001).

A similar pattern was observed for *IL18* expression (Figure 6E), which increased significantly following TLR4 blockade in neutrophils stimulated with LPS (10.24 ± 0.629 vs. 2.877 ± 0.384 without blockade, *p* < 0.01) and with *S*. *schenckii* conidia (7.794 ± 1.931 vs. 1.814 ± 0.022 without blockade, *p* < 0.05).

Finally, *IL1B* expression (Figure 6F) increased markedly only after LPS stimulation, substantially surpassing the induction by conidia (34.27 ± 9.963 vs. 4.981 ± 1.890, *p* < 0.01). However, the response to conidia showed no significant differences compared to the unstimulated control (1.081 ± 0.303, *p* > 0.05). Nevertheless, TLR4 blockade significantly reduced *IL1B* expression in LPS-challenged neutrophils (12.32 ± 2.279, *p* < 0.05), whereas in conidia-stimulated cells this effect did not reach statistical significance (3.036 ± 0.505, *p* > 0.05).

### 3.6. TLR4 Blockade Does Not Alter IL-8 Secretion in S. schenckii-Stimulated Neutrophils

Regarding IL-8 secretion, no statistically significant differences were observed between TLR4-blocked and non-blocked neutrophils upon stimulation with either *S*. *schenckii* (1161 ± 126.8 pg/mL vs. 1256 ± 183.6 pg/mL, *p* > 0.05) or LPS (1375 ± 56.74 pg/mL vs. 1399 ± 101.4 pg/mL, *p* > 0.05) following a 2 h incubation period (Figure 7). However, as described above, IL-8 mRNA showed a slight, non-significant increase following TLR4 blockade in neutrophils stimulated with both LPS and the fungal stimulus.

## 4. Discussion

Here, it is demonstrated that both TLR4-mediated recognition and MPO activity participate in the innate immune response of neutrophils against *S*. *schenckii*. Unlike traditional epidemiological approaches to sporotrichosis, our findings help to elucidate the initial cellular mechanisms by which the host attempts to inhibit the infection by this dimorphic fungus. The results suggest that recognition of the fungus by TLR4 may modulate the inflammatory process associated with MPO secretion while simultaneously activating signaling pathways that trigger cytokine secretion to control the infection, positioning this pathway as a potential immunotherapeutic target. However, several findings from this study remain to be quantitatively validated.

Given that there are few studies addressing the innate immune response of neutrophils in sporotrichosis, it was necessary to establish a positive control for TLR4-mediated activation using LPS as a classical agonist. A fundamental methodological requirement was to ensure an adequate population of viable and metabolically active cells [23], evaluated by MTT reduction. At a concentration of 200 ng, neutrophil viability exceeded 90% and was correlated with maximum MPO activity (both intracellular and secreted), showing a slight, non-significant increase in the secreted form that could reflect a pro-inflammatory behavior. A similar behavior has been observed when stimulating mouse peritoneal exudate cells (PECs) with the *N*. *meningitidis* serogroup B LPS, where elevated levels of proinflammatory cytokines and nitric oxide have been documented [24]. On the other hand, it has been reported that the increase in MPO activity induces inflammation in murine models challenged with LPS and can trigger cell death by caspase-1-dependent pyroptosis [25]. Although this study observed a downward trend in procaspase-1 expression following stimulation with LPS or *S*. *schenckii* conidia—accompanied by the presence of bands corresponding to the active form of caspase-1—the activation and levels of the latter were not quantified.

It has been demonstrated that, within the TLR family, TLR4 acts as the main regulator of neutrophil survival against stimuli such as commercial LPS; in addition, the use of inhibitors of pathways activated downstream of this receptor induces inhibitor-dependent apoptosis [26]. Due to the impossibility of having TLR4-deficient neutrophils, neutralization conditions were established in such a way that allowed the detection of the receptor protein and its cellular immunolocalization, maintaining viability during the analyzed interaction period [26]. Likewise, it is important to note that in this study an antibody derived from the HTA125 clone was used, which has been reported to have neutralizing effects on TLR4 [26,27,28,29]. This ensured that the innate immune response evaluated against *S*. *schenckii* conidia was genuine and not a consequence of cell death, a bias previously documented in other cell types [30]. Although the results presented with heat-killed conidia were used as methodological validation to discard effects attributable to fungal viability, a slight, non-significant increase in metabolic activity was observed upon blocking TLR4. It is likely that the structural modifications suffered by the fungal cell during thermal treatment alter the host response, as has been described in ex vivo assays with mouse splenocytes [31].

Notably, the neutrophils analyzed co-sedimented with the erythrocyte fraction during isolation from peripheral blood of healthy donors, reaching over 90% purity using a protocol previously validated by our group [20]. These features identify these cells as conventional or high-density neutrophils (HDNs), distinguishing them from low-density neutrophils (LDNs), which co-isolate with the mononuclear fraction and predominate in pathological contexts [32,33]. Therefore, it can be inferred that the interaction analyzed at 2 h was between HDNs and *S*. *schenckii*. Although TLR4 and MPO were immunolocalized and NET formation was observed in neutrophils challenged with the fungus, confirming the presence of MPO within these structures, quantitative assays will be necessary to evaluate this process in greater depth using the proposed model. Nevertheless, our findings align with previous reports on *S*. *globosa* indicating that NET production (and its abolition following TLR4 blockade) depends on the specific neutrophil subpopulation involved (HDN vs. LDN) [16]. Furthermore, no NETs were observed following stimulation with LPS, a known NETosis inducer, albeit less potent than phorbol 12-myristate 13-acetate (PMA) [34].

TLR4 blockade led to a reduction—albeit non-significant—in the phagocytic capacity of neutrophils, a finding consistent with the established relevance of this receptor in *S*. *schenckii* recognition by macrophages [12]. Similarly, when TLR4 in human monocyte-derived macrophages is blocked, their phagocytic capacity against *S*. *schenckii* remains unaltered (except when facing mutants in the fungal wall composition), and cytokine secretion (TNF-α, IL-1β, IL-6, IL-17, and IL-23) in human peripheral blood mononuclear cells is unaffected; notably, a distinct response is observed against *S*. *brasiliensis* [35]. Upon TLR4 blockade, other PRRs—such as TLR2, dectin-1, or the mannose receptor—may compensate for fungal recognition, as their involvement in detecting *S*. *schenckii* has been previously demonstrated in other cell types [36]. This alternative recognition pathway offers a research opportunity to further explore the slight decrease in fungal viability observed here.

Following TLR4 blockade, elevated intracellular MPO levels point to altered enzyme accumulation. While MPO generates antimicrobial HOCl [37], assessing phagosomal localization and oxidative activity is necessary to confirm enhanced antifungal function. Although secreted MPO was slightly higher without blockade, potential HOCl-mediated MPO inactivation [38] might be counterbalanced by *S*. *schenckii* catalases [39,40]. Nonetheless, MPO remains vital for clearance in various infections [41]. Intracellular MPO serves as a neutrophil viability marker [23], whereas secreted fractions relate to degranulation and inflammation [42]. Additionally, higher *MPO* mRNA expression in unblocked neutrophils might suggest that compensatory recognition mechanisms sustain the intracellular oxidative response despite reduced early transcription.

TLR4 blockade led to an upregulation of *TLR4* mRNA during the neutrophil–*Sporothrix* interaction, exceeding the transcription levels induced by LPS—a classical TLR4 agonist known to activate pro-inflammatory signaling pathways and cytokine release [43]. In contrast, while LPS stimulation of unblocked neutrophils significantly upregulated *IL1B* mRNA a key promoter of neutrophil recruitment [44], TLR4 blockade during *S*. *schenckii* challenge caused no detectable changes in *IL1B* transcription. These disparities align with evidence indicating that TLR4-dependent cytokine responses vary across experimental models, host cell types, species, and specific *Sporothrix* morphotypes or cell wall components [10,45]. Notably, TLR4 blockade increased *TNF*, *IL18*, and *IL8* mRNA levels relative to unblocked controls, suggesting that these transcriptional adjustments might promote an inflammatory environment. IL-8 signaling induces neutrophil morphological changes, exocytosis, respiratory burst, and receptor upregulation; thus, sustained IL-8 pathway activation could account for the observed *TLR4* transcript upregulation following receptor blockade [46]. Although secreted IL-8 protein levels remained unchanged at 2 h regardless of TLR4 status, this delayed kinetic response mirrors observations in conidia-challenged keratinocytes, where significant IL-8 secretion requires at least 8 h [47].

The changes observed in cytokine mRNA expression imply that the experimental conditions modulate cytokine gene transcription. However, increased transcript abundance should not be interpreted as direct evidence of increased cytokine production or enhanced inflammatory activity. Cytokine expression is regulated at multiple levels, including mRNA stability, translation, intracellular processing, secretion, and protein degradation, and therefore transcript levels may not directly correlate with extracellular protein concentrations. This distinction is particularly evident for IL-8 in the present study, because *IL8* mRNA expression and IL-8 secretion did not display the same pattern among experimental conditions. These findings suggest that additional post-transcriptional or post-translational regulatory mechanisms may influence the final amount of IL-8 released by the cells.

Furthermore, it has been documented that TNF-α secretion by TLR4-deficient peritoneal macrophages from mice decreases during interaction with *S*. *schenckii* [10]. In keratinocytes, which express low levels of TLR4 and are challenged with conidia, the secretion levels of this cytokine are low and even lower when challenged with yeasts [47]. In tissue homogenates from dectin-1 and -2-deficient mice, no changes in the concentration of this cytokine were observed [48]. However, overexpression of *TNF* mRNA with neutrophils was found when the receptor was blocked. Likewise, this behavior was observed with *IL18* mRNA, which encodes the inflammatory cytokine mainly secreted by dendritic cells and Langerhans cells through the IL-18/IL-2/NF-ĸB axis when challenged with *S*. *globosa*; in addition, it was found that it can participate in the polarization process toward a Th2 profile associated with the sporotrichosis chronicity [47]. However, notably, an increase in the transcriptional modulation of IL-18 was observed in unstimulated neutrophils treated with the neutralizing antibody, a finding limited exclusively to this cytokine. This observation might be explained by the possibility that the anti-TLR4 neutralizing antibody (clone HTA125), as an IgG2a isotype, binds nonspecifically to Fcγ receptors [29] and consequently acts as an agonist that activates neutrophils. It has been demonstrated that the Fc region of mouse IgG2a—and therefore that of the HTA125 antibody—can be recognized by Fcγ receptors [49], forming immune complexes that activate neutrophils in the absence of infection [50], which could trigger the synthesis, expression, and release of IL-18 by human neutrophils [51]. Consequently, future comparative experiments will be necessary to distinguish specific TLR4 effects from antibody-related effects by including an isotype control, an aspect that represents a limitation of the present study.

The observed behavior in neutrophils with blocked TLR4 and challenged with LPS was similar for the expression of *TNF*, *IL18*, and *IL8*. The recorded heterogeneous cellular responses following TLR4 blockade suggest that some of the effects induced by fungal recognition may not be exclusively dependent on TLR4 signaling. As previously mentioned, fungal components can be recognized by multiple pattern-recognition receptors, including TLR2, Dectin-1, the mannose receptor, and other PRRs. Therefore, residual signaling through TLR4-independent pathways could potentially contribute to the preservation of some cellular responses despite TLR4 inhibition; however, the activity of these alternative receptors was not directly evaluated here. Consequently, their contribution cannot be established from our results, and compensatory signaling through other PRRs should be considered as a hypothesis rather than a demonstrated mechanism. The observed heterogeneity is compatible with the possibility that multiple recognition pathways participate in the cellular response, but additional experiments are required to identify the involved receptors and to determine their relative contribution.

The findings of this study suggest that TLR4 contributes to the modulation of human neutrophil responses to *S*. *schenckii* conidia. TLR4 blockade was associated with changes in MPO-related parameters and in selected inflammatory mediators transcription and secretion processes; however, these responses were heterogeneous and were not accompanied by a significant effect on fungal viability. Therefore, the data shown here support a modulatory role for TLR4 rather than demonstrating an exclusive or definitive TLR4-dependent mechanism of fungal recognition. The inflammatory mediators evaluated in this study may contribute to our understanding of the coordination of innate immune responses and interactions with adaptive immunity. For example, TNF-α can promote macrophage activation and enhance functions such as phagocytosis and antigen presentation [48]. Nevertheless, changes observed at the transcriptional level should not be interpreted as direct evidence of increased protein production or functional inflammatory activity, unless supported by the respective protein-level measurements. Overall, the results show that TLR4 may participate in shaping the neutrophil response to *S*. *schenckii* conidia, although additional recognition pathways are likely to contribute to this process. Further studies are required to determine the specific contribution of TLR4 signaling, to clarify the MPO-dependent oxidative mechanisms, to evaluate the role of NETs and caspase-1 activation, and to investigate the possible involvement of other pattern-recognition receptors under conditions of TLR4 blockade. The relevance of this work is that it provides information related to human neutrophils in response against *S*. *schenckii*.

## Figures and Tables

**Figure 1:**
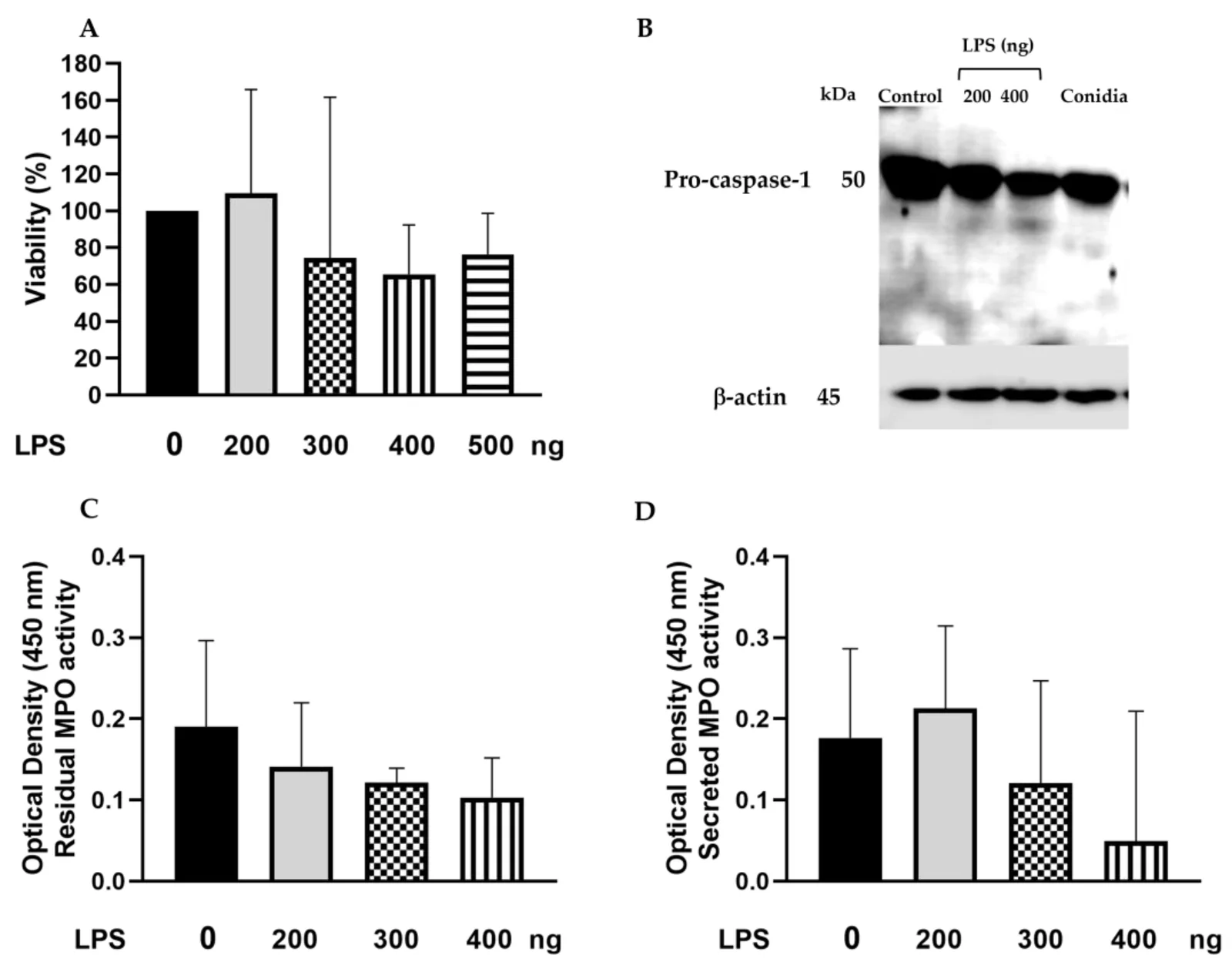
Effect of LPS on human neutrophil activation. Neutrophils were stimulated with different concentrations of LPS (0, 200, 300, 400 and 500 ng/250 µL). (**A**) Cell viability. (**B**) Representative blots of pro-caspase-1 and β-actin (loading control) assessed by Western blot of protein extracts from neutrophils challenged with LPS and conidia of *S*. *schenckii*. Full-length uncropped blots are provided in the Supplementary Materials (Figure S1). (**C**) Residual (intracellular) and (**D**) Secreted MPO activity in the supernatant MPO activity (right). Data are presented as mean ± SEM of *n* = 2 independent experiments (each experiment performed with neutrophils pooled from two different healthy donors). Statistical differences were determined by one-way ANOVA followed by Tukey’s multiple comparisons test.

**Figure 2:**
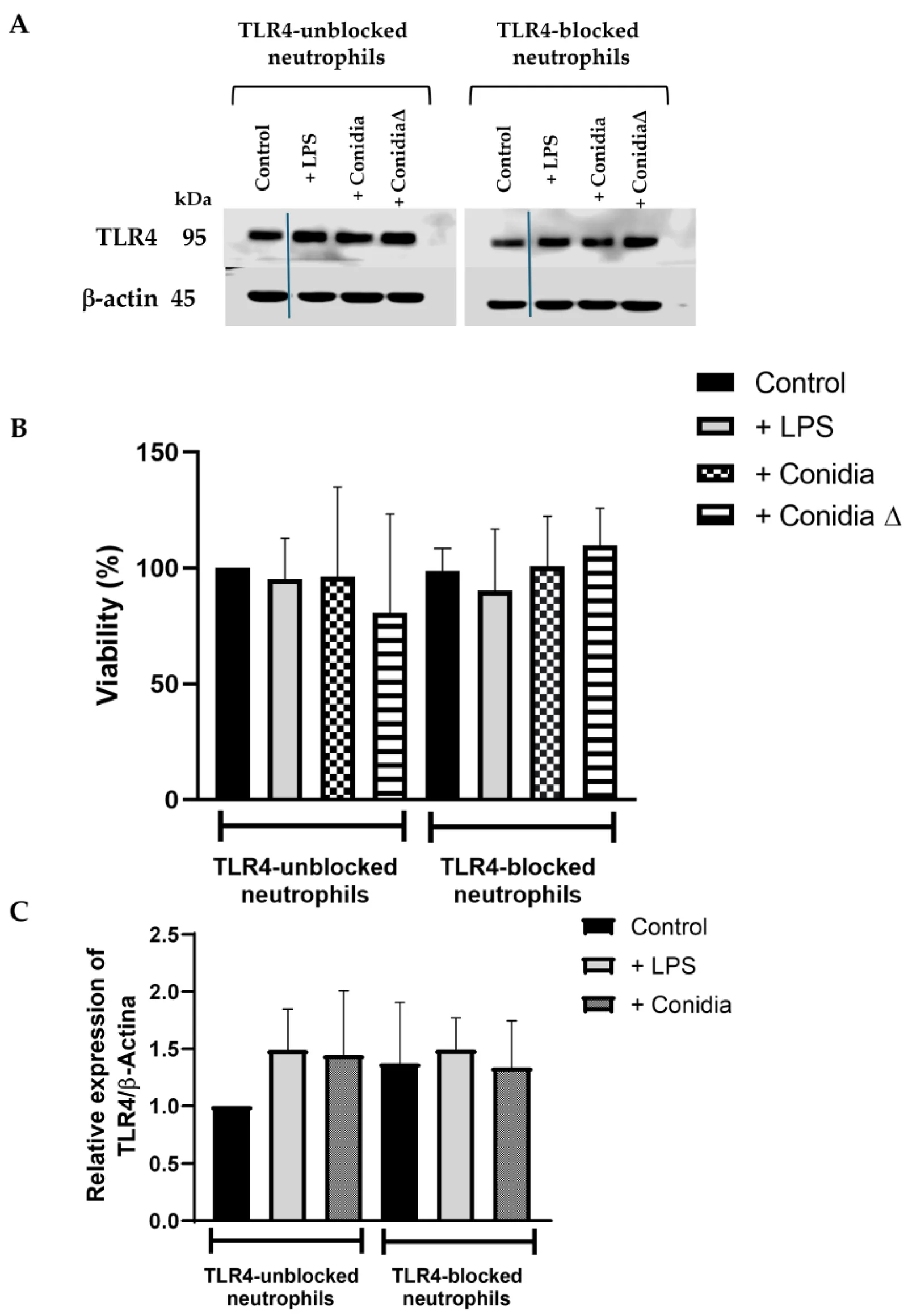
Effect of TLR4 blockade on human neutrophils. (**A**) Representative Western blots showing TLR4 and β-actin (loading control) expression in protein extracts from neutrophils challenged with LPS (200 ng/250 μL RPMI-1640), viable conidia, and heat-killed conidia (Δ). Full-length uncropped blots are provided in the Supplementary Materials (Figure S2). (**B**) Cell viability percentage. (**C**) Densitometric quantification of TLR4 expression normalized to β-actin (arbitrary units). Data are expressed as mean ± SEM of *n* = 3 independent experiments (each experiment performed with neutrophils pooled from two different healthy donors). Statistical significance was determined using one-way ANOVA followed by Tukey’s post hoc test.

**Figure 3:**
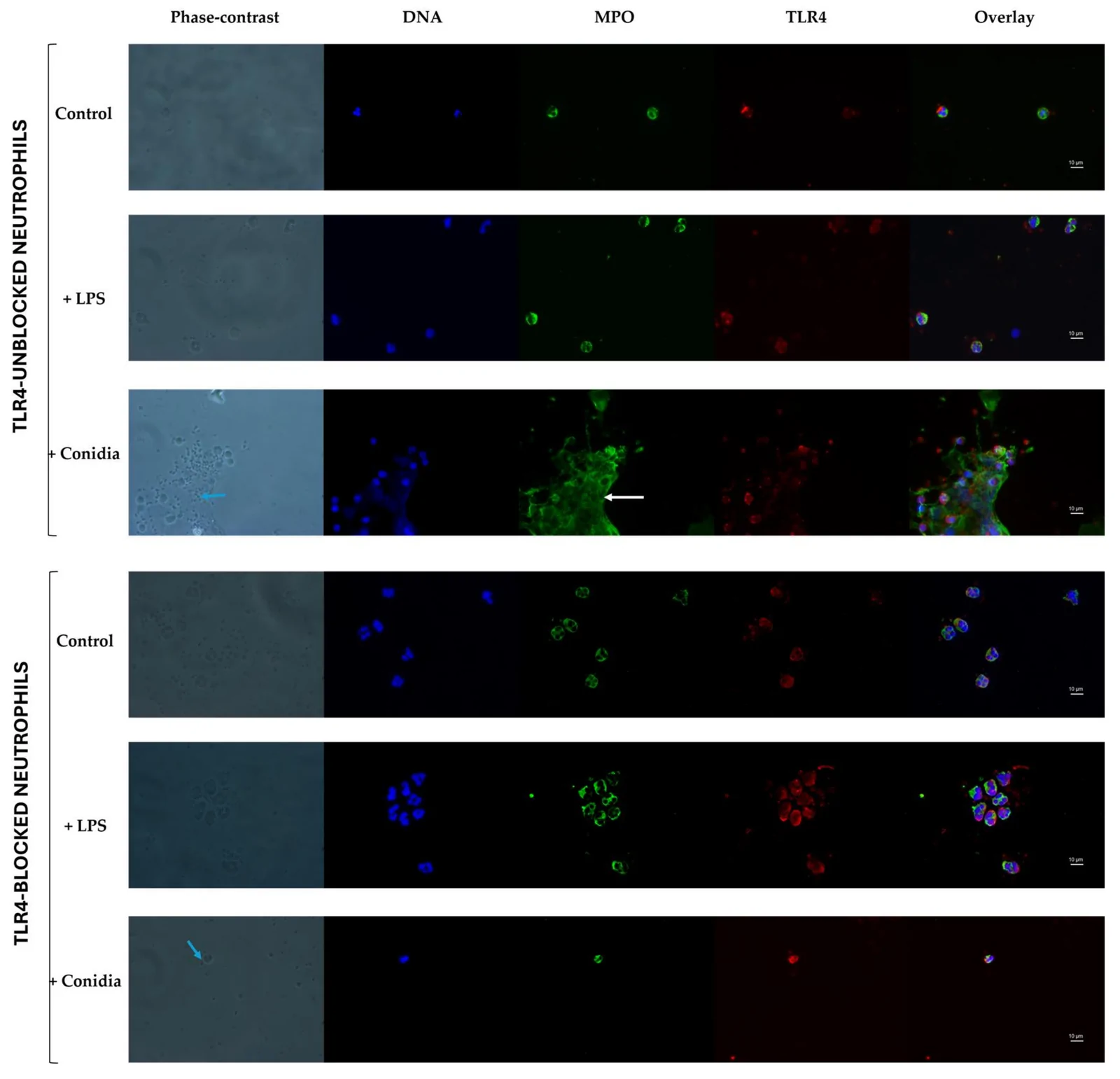
Representative immunofluorescence images of neutrophils with or without TLR4 blockade, stimulated with LPS (200 ng/250 μL RPMI-1640) or *S*. *schenckii* conidia for 2 h. Cells were stained for MPO (green) and TLR4 (red); nuclei were counterstained with Hoechst (blue). Merged images are shown on the right panel. Scale bar = 10 µm (400× magnification). Blue arrows indicate conidia. White arrows indicate representative NETs-like structures.

**Figure 4:**
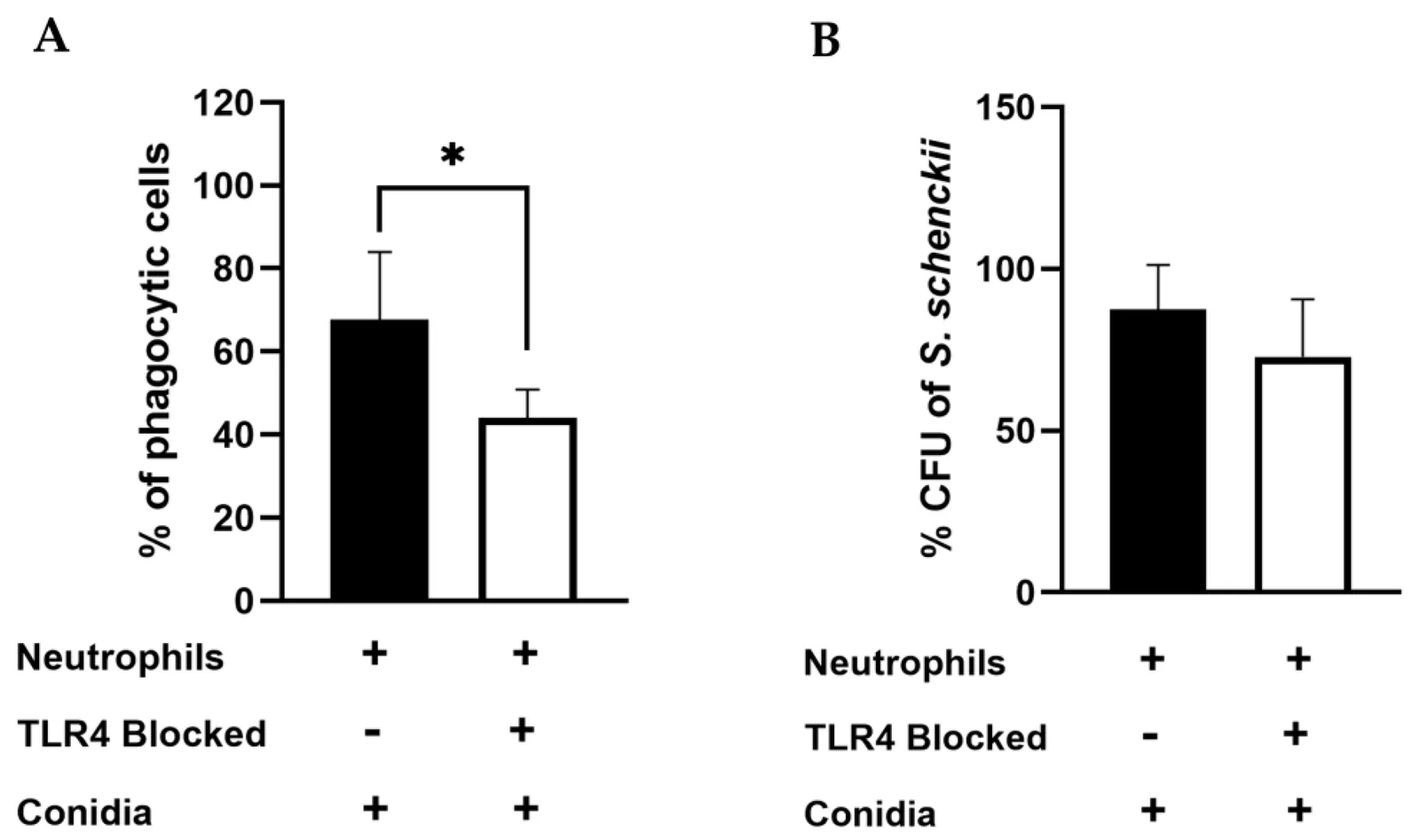
Effect of TLR4 blockade on human neutrophil phagocytic activity and *S*. *schenckii* viability. (**A**) Percentage of cells with phagocytic activity. (**B**) Fungal viability was expressed as colony-forming units (CFU) following interaction of *S*. *schenckii* with human neutrophils. Data are presented as mean ± SEM of *n* = 3 independent experiments (each experiment performed with neutrophils pooled from two different healthy donors). Statistical significance was determined using a two-tailed Student’s *t*-test (* *p* < 0.05 vs. non-blocked TLR4 control group).

**Figure 5:**
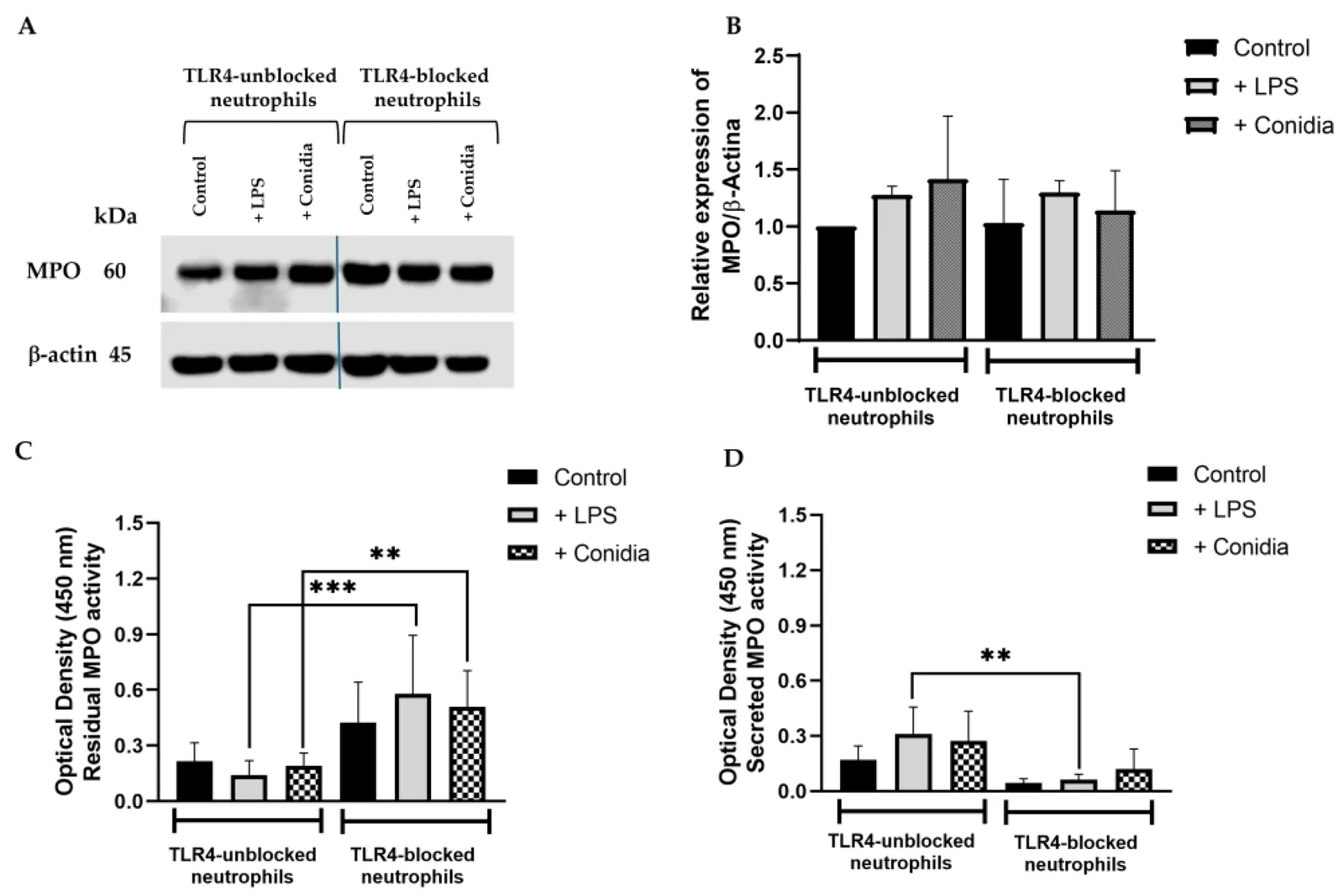
Effect of TLR4 blockade on myeloperoxidase expression and activity in neutrophils challenged with *S*. *schenckii*. (**A**) Representative immunoblots of MPO and β-actin (loading control) analyzed by Western blot from neutrophil protein extracts (top), and (**B**) quantification of MPO expression normalized to actin in arbitrary units (bottom). Full-length uncropped blots are provided in the Supplementary Materials (Figure S3). (**C**) Residual (intracellular) MPO activity. (**D**) MPO activity secreted into the supernatant. Data are presented as mean ± SEM of *n* = 3 independent experiments (each experiment performed with neutrophils pooled from two different healthy donors). Statistical differences were determined by one-way ANOVA followed by Tukey’s multiple comparisons test; Statistical significance is indicated as follows: *** *p* < 0.001 and ** *p* < 0.01.

**Figure 6:**
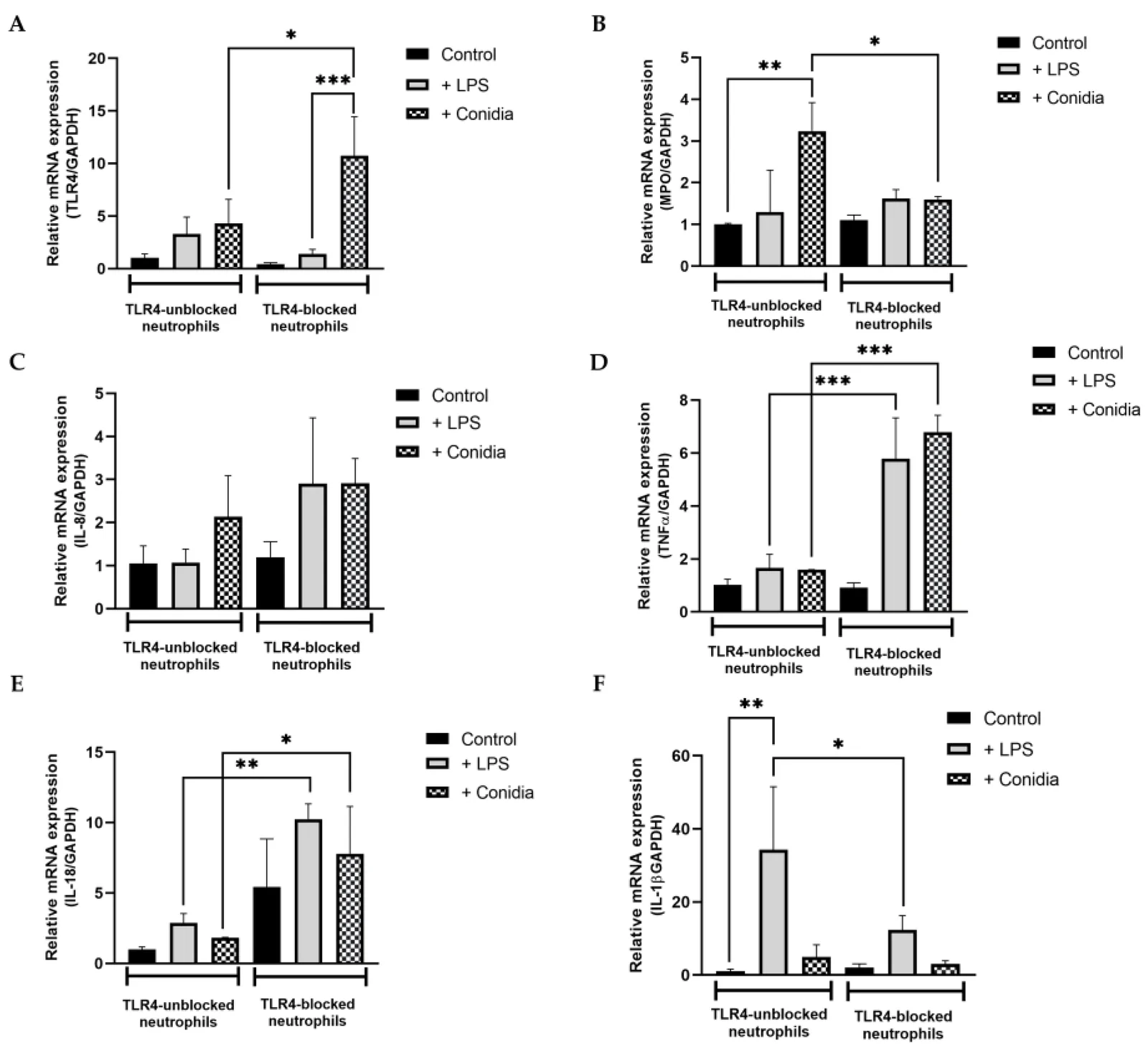
Effect of TLR4 blockade on gene expression and cytokine response in neutrophils stimulated with *S*. *schenckii* conidia. Neutrophils were pretreated with or without a neutralizing anti-TLR4 antibody prior to stimulation with *S*. *schenckii* conidia for 2 h. Relative mRNA expression levels of (**A**) *TLR4*, (**B**) *MPO*, (**C**) *IL8*, (**D**) *TNF*, (**E**) *IL18*, and (**F**) *IL1B* were evaluated by RT-qPCR and normalized using *GAPDH* as an internal control. Data are presented as mean ± SEM of *n* = 2 independent experiments (each experiment performed with neutrophils pooled from two different healthy donors). Statistical differences were determined by one-way ANOVA followed by Tukey’s multiple comparisons test; Statistical significance is indicated as follows: *** *p* < 0.001, ** *p* < 0.01 and * *p* < 0.05.

**Figure 7:**
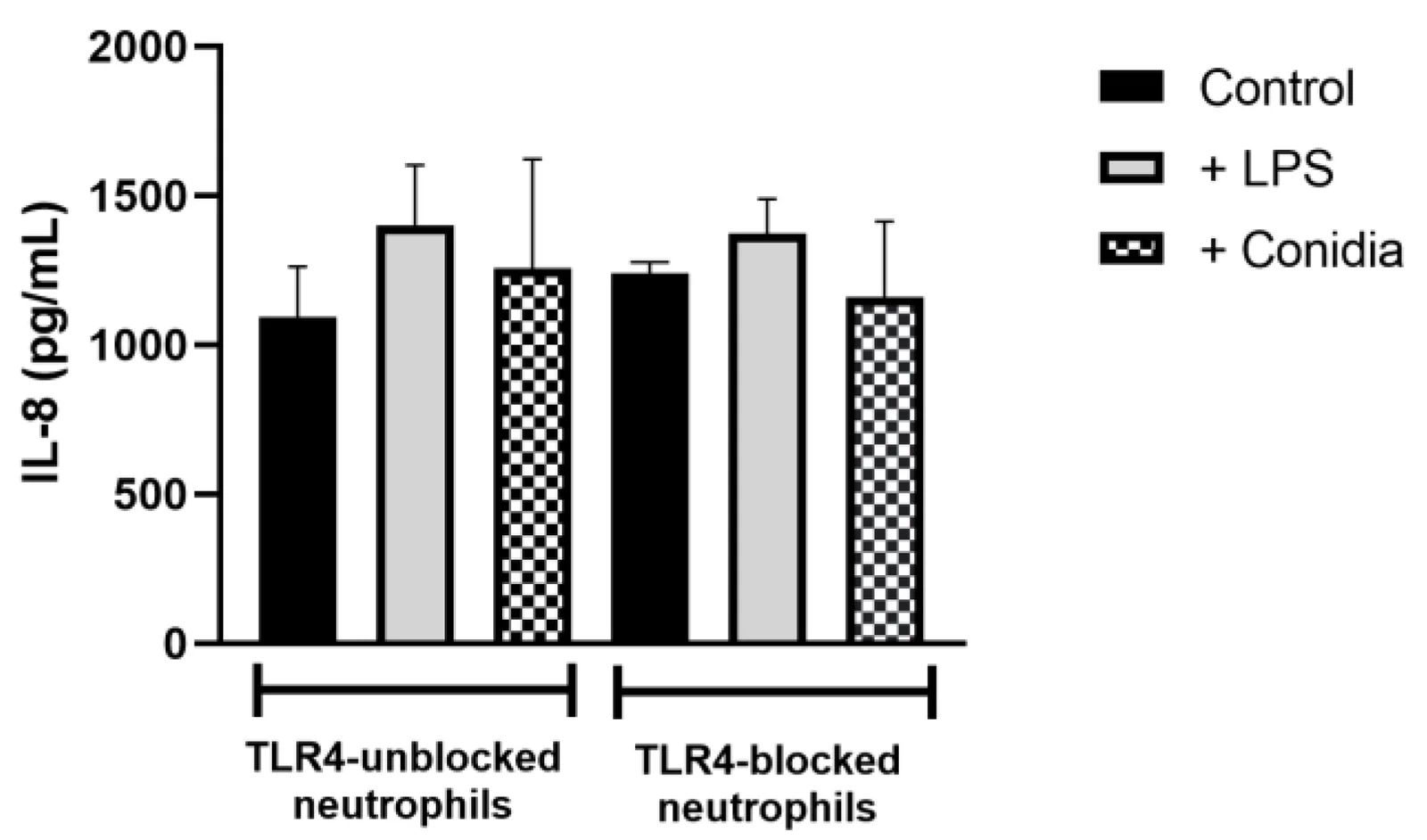
Effect of TLR4 blockade on IL-8 secretion in human neutrophils stimulated with *S*. *schenckii***.** Neutrophils were pre-incubated with or without an anti-TLR4 blocking antibody and subsequently challenged with *S*. *schenckii* for 2 h. IL-8 concentrations in culture supernatants were quantified by ELISA. Data are presented as mean ± SEM of *n* = 2 independent experiments (each experiment performed with neutrophils pooled from two different healthy donors). Statistical differences were determined by one-way ANOVA followed by Tukey’s multiple comparisons test. No statistically significant differences were found between TLR4-blocked and control cells (*p* > 0.05).

**Table 1:** Oligonucleotide sequences used in this study.

Target Gene	Sequence (5′→3′)	Access Number	Product Size (bp)
*IL8*	Fw: GCCAAGGAGTGCTAAAGAAC	NM_000584.4	200
	Rv: AACTTCTCCACAACCCTCTG		
*IL1B*	Fw: GGAATTTGAGTCTGCCCAGT	NM_000576.2	206
	Rv: CTTTCTGTTCCCTTTCTGCC		
*IL18*	Fw: TTTCAGAGAAGTGTCCCAGG	NM_001562.4	137
	Rv: TCTATCCCCCAATTCATCCT		
*TNF*	Fw: ACATCCAACCTTCCCAAACGCC	NM_000594.4	111
	Rv: TTCCGACCCTAAGCCCCCAA		
*TLR4*	Fw: CACTCTGTCACTTTGTCACTCG	NM_138554.5	183
	Rv: GTCATCCCACTTCCTTCCTG		
*MPO*	Fw: GCTGGCAAGGGGATAAGAGA	NM_000250.2	120
	Rv: CTTCTCCTGGGCTGCTCAAT		
*GAPDH*	Fw: TGGTCTCCTCTGACTTCAACA	NM_002046.7	117
	Rv: AGCCAAATTCGTTGTCATACC		

## Data Availability

Data may be shared and should be requested from the corresponding author.

## Supplementary Materials

The following supporting information can be downloaded at: https://www.mdpi.com/article/10.3390/jof12100779/s1, Figure S1. Original, uncropped Western blot membranes for the immunoblots in Figure 2A. Representative immunoblots of TLR4 and β-actin (loading control) analyzed by Western blot from neutrophil protein extracts. Neutrophils (3 × 10^6^ cells) treated with or without a neutralizing anti-TLR4 antibody (1:100; Mouse Monoclonal Antibody (HTA125), Invitrogen, Thermo Fisher Scientific, Waltham, MA, USA; MA5-16719) were subjected to the following condition during 2 h at 37 °C: Control: Neutrophils without stimuli; +PMA: Neutrophils stimulated with phorbol 12-myristate 13-acetate (PMA, 1 μg/mL, Sigma-Aldrich, St. Louis, MO, USA); +LPS: Neutrophils stimulate with lipopolysaccharide (200 ng/250 μL RPMI-1640); +Conidia: Neutrophils stimulated with viable *S*. *schenckii* conidia (15 × 10^6^ cells); +ConidiaΔ: Neutrophils stimulated with heat-killed *S*. *schenckii* conidia (conidial suspensions were incubated in a water bath at 60 °C for 2 h); Figure S2: Original, uncropped Western blot membranes for the immunoblots in Figure 2A. Representative immunoblots of TLR4 and β-actin (loading control) analyzed by Western blot from neutrophil protein extracts. Neutrophils (3 × 10^6^ cells) treated with or without a neutralizing anti-TLR4 antibody (1:100; Mouse Monoclonal Antibody (HTA125), Invitrogen, Thermo Fisher Scientific, Waltham, MA, USA, MA5-16719) were subjected to the following condition during 2 h at 37 °C: Control: Neutrophils without stimuli; +PMA: Neutrophils stimulated with phorbol 12-myristate 13-acetate (PMA, 1 μg/mL, Sigma-Aldrich, St. Louis, MO, USA); +LPS: Neutrophils stimulated with lipopolysaccharide (200 ng/250 μL RPMI-1640); +Conidia: Neutrophils stimulated with viable *S*. *schenckii* conidia (15 × 10^6^ cells); +ConidiaΔ: Neutrophils stimulated with heat-killed *S*. *schenckii* conidia (conidial suspensions were incubated in a water bath at 60 °C for 2 h); Figure S3: Original, uncropped Western blot membranes for the immunoblots in Figure 5A. Representative immunoblots of MPO and β-actin (loading control) analyzed by Western blot from neutrophil protein extracts. Neutrophils (3 × 10^6^ cells) treated with or without a neutralizing anti-TLR4 antibody (1:100; Mouse Monoclonal Antibody (HTA125), Invitrogen, Thermo Fisher Scientific, Waltham, MA, USA, MA5-16719) were subjected to the following condition during 2 h at 37 °C: Control: Neutrophils without stimuli; +LPS: Neutrophils stimulated with lipopolysaccharide (200 ng/250 μL RPMI-1640); +Conidia: Neutrophils stimulated with viable *S*. *schenckii* conidia (15 × 10^6^ cells); +ConidiaΔ: Neutrophils stimulated with heat-killed *S*. *schenckii* conidia (conidial suspensions were incubated in a water bath at 60 °C for 2 h).

## Abbreviations

The following abbreviations are used in this manuscript:

MPO	Myeloperoxidase
TLR4	Toll-Like Receptor 4
LPS	Lipopolysaccharide
MTT	Methyl Thiazolyl Tetrazolium
TMB	3,3′,5,5′-tetramethylbenzidine
RPMI-1640	Roswell Park Memorial Institute)-1640 medium
RIPA	Radioimmunoprecipitation assay buffer
SDA	Sabouraud Dextrose Agar
SDA-A	Sabouraud dextrose agar supplemented with antibiotics (cycloheximide and chloramphenicol)
CFUs	Colony-Forming Units
OD	Optical Density
FBS	Fetal Bovine Serum
BSA	Bovine Serum Albumin
IFN	Interferon
IL	Interleukin
TNF	Tumor necrosis factor
NK-κB	Nuclear Factor kappa-light-chain-enhancer of activated B cells
PAMP	Pathogen-associated molecular patterns
PRR	Pattern recognition receptors
NETs	Neutrophil Extracellular Traps
