# Supplementary material for: TLR4 Regulates the Proinflammatory Response and Myeloperoxidase Activity in Neutrophils During Interaction with Sporothrix schenckii
Source: J Fungi (Basel). 2026 Oct 17;12(10):779. doi: 10.3390/jof12100779 (PMC13706431; doi:10.3390/jof12100779)
Supplement: Supplementary file 1 [file jof-12-00779-s001.zip › jof-4564699-supplementary.pdf]

Supplementary Material

TLR4 Regulates the Proinflammatory Response and Myeloperoxidase Activity in Neutrophils During Interaction with *Sporothrix schenckii*

Yolanda Romo-Lozano [1,*], Aylin Mayela Avila-Colis [1], Daniel Cervantes-García [1,2], Rodolfo González-Segovia [1] and Estela Ruiz-Baca [3]

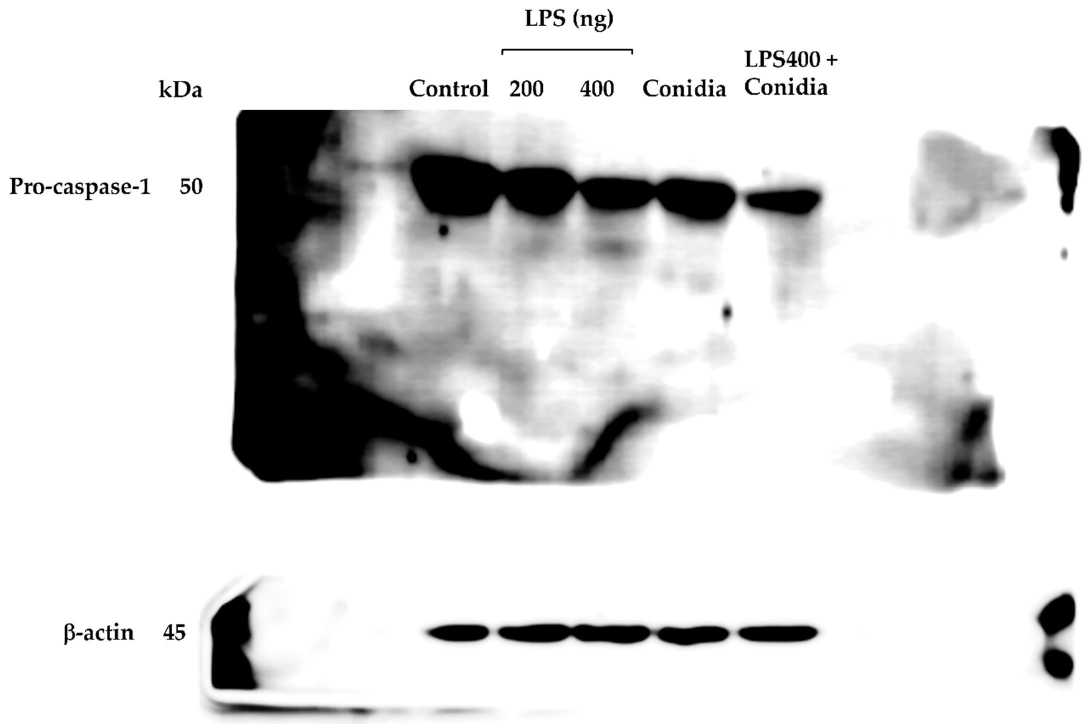

Figure S1. Original, uncropped Western blot membranes for the immunoblots in Figure 2A. Representative immunoblots of TLR4 and β-actin (loading control) analyzed by Western blot from neutrophil protein extracts. Neutrophils (3 x 10^6 cells) treated with or without a neutralizing anti-TLR4 antibody (1:100; Mouse Monoclonal Antibody (HTA125), Invitrogen, Thermo Fisher Scientific, Waltham, MA, USA, MA5-16719) were subjected to the following condition during 2 h at 37 °C: Control: Neutrophils without stimuli; +PMA: Neutrophils stimulated with phorbol 12-myristate 13-acetate (PMA, 1 µg/mL, Sigma-Aldrich, St. Louis, MO, USA); +LPS: Neutrophils stimulate with lipopolysaccharide (200 ng/250 µL RPMI-

1640); +Conidia: Neutrophils stimulated with viable *S. schenckii* conidia (15 x 10^6 cells); +ConidiaΔ: Neutrophils stimulated with heat-killed *S. schenckii* conidia (conidial suspensions were incubated in a water bath at 60 °C for 2 h).

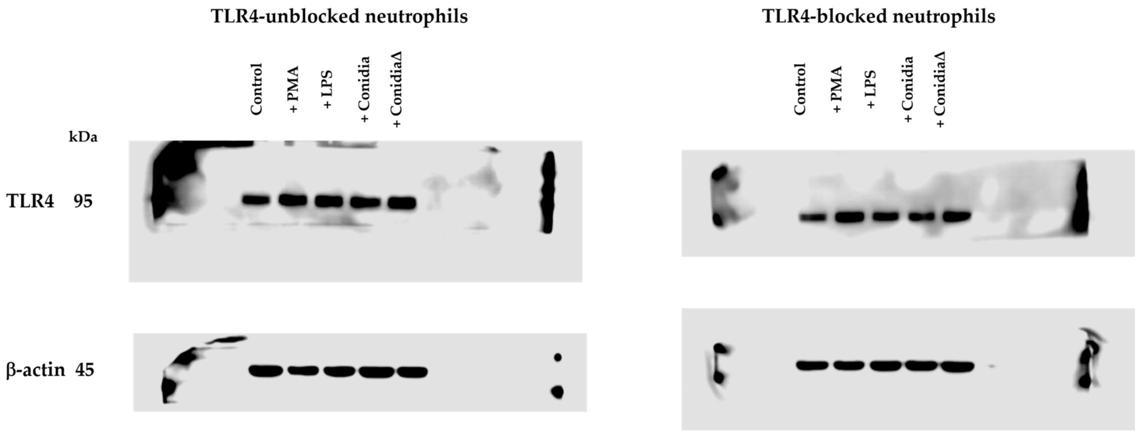

Figure S2. Original, uncropped Western blot membranes for the immunoblots in Figure 2A. Representative immunoblots of TLR4 and β-actin (loading control) analyzed by Western blot from neutrophil protein extracts. Neutrophils (3 x 10^6 cells) treated with or without a neutralizing anti-TLR4 antibody (1:100; Mouse Monoclonal Antibody (HTA125), Invitrogen, Thermo Fisher Scientific, Waltham, MA, USA, MA5-16719) were subjected to the following condition during 2 h at 37 °C: Control: Neutrophils without stimuli; +PMA: Neutrophils stimulated with phorbol 12-myristate 13-acetate (PMA, 1 μg/mL, Sigma-Aldrich, St. Louis, MO, USA); +LPS: Neutrophils stimulate with lipopolysaccharide (200 ng/250 μL RPMI-1640); +Conidia: Neutrophils stimulated with viable *S. schenckii* conidia (15 x 10^6 cells); +ConidiaΔ: Neutrophils stimulated with heat-killed *S. schenckii* conidia (conidial suspensions were incubated in a water bath at 60 °C for 2 h).

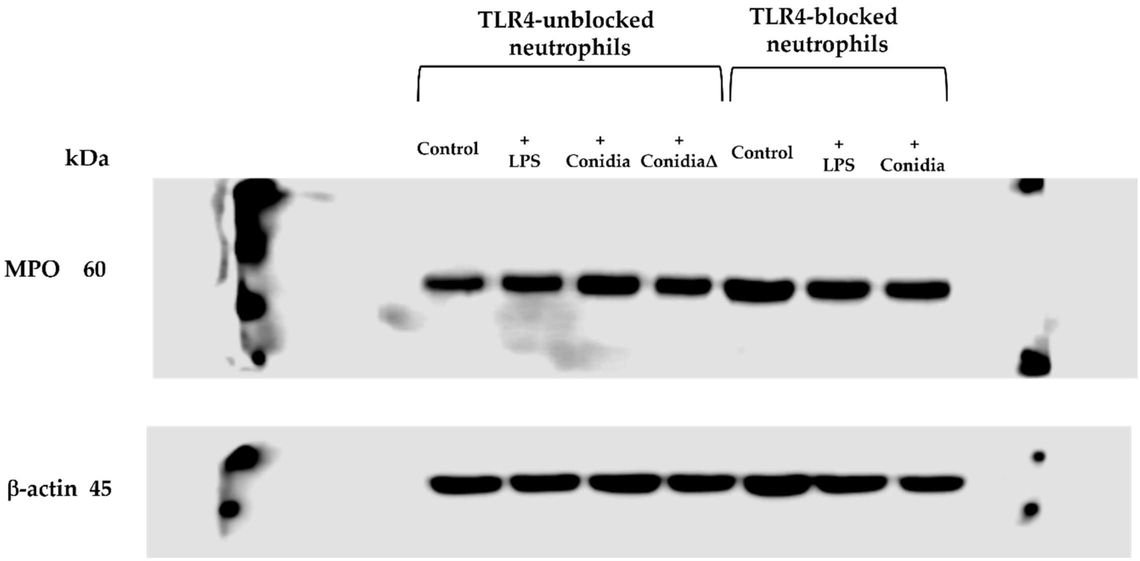

Figure S3. Original, uncropped Western blot membranes for the immunoblots in Figure 5A. Representative immunoblots of MPO and β-actin (loading control) analyzed by Western blot from neutrophil protein extracts. Neutrophils (3 x 10^6 cells) treated with or without a neutralizing anti-TLR4 antibody (1:100; Mouse Monoclonal Antibody (HTA125), Invitrogen, Thermo Fisher Scientific, Waltham, MA, USA, MA5-16719) were subjected to the following condition during 2 h at 37 °C: Control: Neutrophils without stimuli; +LPS: Neutrophils stimulated with lipopolysaccharide (200 ng/250 μL RPMI-1640); +Conidia: Neutrophils stimulated with viable *S. schenckii* conidia (15 x 10^6 cells); +ConidiaΔ: Neutrophils stimulated with heat-killed *S. schenckii* conidia (conidial suspensions were incubated in a water bath at 60 °C for 2 h).
